# Multisensory integration in virtual interactions with distant objects

**DOI:** 10.1038/s41598-019-53921-9

**Published:** 2019-11-22

**Authors:** Wladimir Kirsch, Wilfried Kunde

**Affiliations:** Department of Psychology, University of Würzburg, Würzburg, Germany

**Keywords:** Human behaviour, Health sciences

## Abstract

Statistically optimal integration of multimodal signals is known to take place in direct interactions with environmental objects. In the present study we tested whether the same mechanism is responsible for perceptual biases observed in a task, in which participants enclose visual objects by manually controlled visual cursors. We manipulated the relative reliability of visual object information and measured the impact of body-related information on object perception as well as the perceptual variability. The results were qualitatively consistent with statistically optimal sensory integration. However, quantitatively, the observed bias and variability measures systematically differed from the model predictions. This outcome indicates a compensatory mechanism similar to the reliability-based weighting of multisensory signals which could underlie action’s effects in visual perception reported in diverse context conditions.

## Introduction

Physical interactions with environmental objects consist of body movements that are directed towards those objects. These movements are typically controlled by visual information, but provide body-related information in the course of moving as well. For example, when we grab a cup of coffee we see and feel the cup being grabbed. In this case, both visual and body-related signals provide information about the same object, i.e. they are redundant. Redundant sensory signals are integrated in a statistically optimal way taking signal reliability into account^1,2^. One consequence of this process is a mutual perceptual attraction between them when a sensory discrepancy is introduced^3^.

Now consider other types of action such as grasping an object with a tool or throwing a basketball into a basket. In contrast to natural grasping, the body-related movement effects are spatially separated from the environmental effects. This spatial gap is bridged by mechanical or electronic devices or by natural forces like gravity. However, given stable environmental conditions there is still a systematic relationship between what is felt and what is seen. Changing the kind of ball throwing and thus the associated body feeling, necessarily changes the visually sensed ball trajectory. Thus, information provided by the body refers to the same event as information about the ball trajectory. In other words, body-related and environmental action effects can be considered, at least to some extent, as redundant like in natural grasping. Against this background it is not surprising that mutual perceptual biases are reported with a spatial separation of body-related and visual action effects when a conflict between them is introduced. For example, the direction of a hand movement performed on a horizontal plane attracts the perceived direction of the visual cursor displayed in the fronto-parallel plane and, vice versa, the movement direction of the cursor attracts the perceived direction of the hand movement when the visual movement direction is misaligned with respect to the actual movement direction^4,5^. This and similar findings suggest that body-related and visual action effects are integrated in spite of a clear spatial separation of their origin in a similar fashion like in direct object interactions^6–9^.

Influences of action on perception are not limited to the perception of objects or events directly caused by the body movement, such as mouse cursor, but can comprise other environmental objects as well. Consider, e.g., a basketball player again whose goal is to hit the basket. There is not only a relationship between the body movement and the ball trajectory, but also between the body movement and the basket itself. For example, the egocentric distance to the basket can be defined based on visual cues as well as on body-related cues such as on the strength of the muscular force pulse, necessary to reach the basket. The situation in tool use is perhaps more obvious. In reaching for an object with a stick held in the hand, e.g., the distance to the object can not only be visually sensed but also bodily felt when the tool geometry is taken into account. Thus, redundancy exists not only between the visual and body-related feedback of object-oriented actions, but also between the visual information of a target object and body-related information of corresponding object-oriented actions. It is thus conceivable that visual information of target objects and body-related information from corresponding object-oriented actions become subject to multisensory integration. If so, then changing the relation between both signals should result in mutual perceptual biases.

There are in fact a lot of studies which either introduced or utilized such a kind of crossmodal conflict and which report effects of real or anticipated body-related feedback on visual perception of external objects in diverse contexts (see^10,11^ for reviews and^12,13^ for critics and controversies). Distant objects, e.g., appear closer when they are manipulated by a tool^14^. We recently reasoned that these and similar observations might be a consequence of sensory integration of redundant signals as outlined above^15^. Using a task in which participants were to enclose a visual target object with manually controlled cursors we demonstrated that actions’ effects in visual perception are accompanied by the effects of visual signals on body perception as predicted by the multisensory approach.

Such indications of multisensory integration of body-related signals and visual information relating to a distant object being a goal of body’s movement are not entirely new. Visual-haptic integration between the size of an object and hand opening has been reported for grasping using plier- and tong-like tools^16–18^. The new and important aspect of our study was that indices of sensory integration were present in the absence of any explicit visual cues, such as a tool, bridging the spatial distance between the body and the object. Thus, our study suggests the applicability of optimal sensory integration to virtually all interactions with external objects.

**Figure 1:**
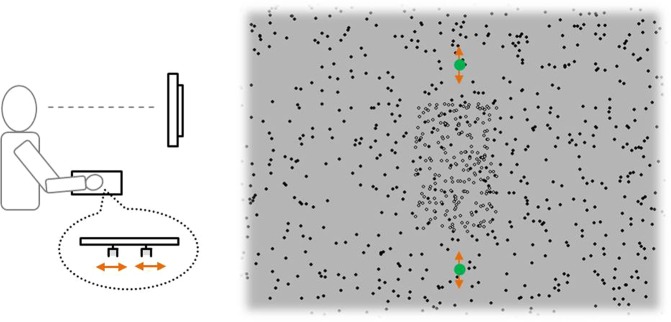
The experimental setup (left) and stimuli (right) used in the present study. The items are not to scale. See main text for further details.

This claim should be considered with caution, however, not least because perceptual biases alone are not sufficient to demonstrate optimal sensory integration^19^. The main goal of the present study was thus to more directly test virtual interactions with distant objects for optimal multisensory integration. For this purpose we adopted the previously used paradigm accordingly. Participants performed three versions of a two-alternative forced-choice discrimination task (2AFC)^20^. In one condition (bimodal condition hereafter), they enclosed two visual objects by a pair of manually controlled visual cursors in succession and judged which stimulus was larger thereafter (see Fig. 1). During one of these actions a discrepancy between the actual finger distance and the cursor distance, and thus the visual object size, was introduced. In another condition (visual condition), the same visual objects were presented and the same judgment was required. However, no finger movements were performed. In a third condition (haptic condition), different finger distances were adopted like in the first condition. However, no visual objects were presented. Additionally, we varied the relative reliability of visual information by manipulating the visual indicators of the target object and its duration.

According to the maximum likelihood estimation (MLE) rule optimal multisensory integration corresponds to a weighted average of unimodal signals with weights being proportional to signal reliability^1^. The basic MLE model does not take into account possible top-down influences (prior assumptions) and assumes full multisensory integration^2^. For the bimodal condition, this approach predicts a perceptual bias towards a more reliable signal and thus a decrease of the bias when this signal becomes less reliable (qualitative predictions hereafter). Quantitatively, the observed bias should not systematically deviate from the predicted one. Also, the precision of discrimination performance should not deviate from the model prediction and should be increased as compared to both unimodal conditions^19^.

## Methods

### General notes on methodical choices

The procedure and design of the present experiment have basically two origins: our previous work on the interplay between perception and action in virtual interactions with distant objects^15,21^, and a seminal work on multisensory integration in grasping^20^. Note that it was not our aim to make the task as similar to natural grasping as possible such as to let the participants experience pressure of their fingers during the interaction with the object (see also Discussion). Rather, we tested whether a previously observed impact of a body-related variable on the visual perception of a distant object can be explained by the MLE model. To do so, some requirements have to be met^19^. In particular, in addition to the main task that enables using of correlated signals from different modalities (see “bimodal” condition below), the experiment should contain single modality conditions which provide the basis for the model predictions for the critical bimodal condition. Moreover, it has been suggested to include different levels of noise (i.e. reliability) in one of the modalities which can provide strong evidence for optimal integration. We adjusted our original paradigm^15,21^ according to this and related guidelines. Thus, except for some details of the task, the methodical approach including the rationale, design and data analyses was basically the same as in the previous research on multisensory integration in grasping^20^.

### Participants

Twenty-four right-handed participants with normal or corrected-to-normal vision were recruited. All participants gave their written informed consent for the procedures and received monetary compensation (40 €) for their participation. The sample included twenty females and four males (*M*_*age*_ = 26, *SD* = 5).

The study was performed in accordance with the Declaration of Helsinki (1964) and has been approved by the local ethics committee (Ethikkommission des Institutes für Psychologie der Humanwissenschaftlichen Fakultät der Julius-Maximilians-Universität Würzburg, GZ 2019-04). Related experiments testing for optimal integration usually use a small number of participants e.g. four in^20^. Following the corresponding guidelines^19^ we initially collected the data of twelve participants. To ensure that the observed pattern of results is reliable we doubled the initial size of the sample. The final sample size ensured a power of 0.95 for effect sizes of d_z_ = 0.7.

### Apparatus

The experimental room was dimly illuminated. A 19′ monitor was in front of the participants at a distance of approximately 68 cm (Fujitsu Siemens P19-1; 1280 × 1024 pixels; 1 pixel = 0.294 mm; 60 Hz). It was centered at approximate eye level. Participant’s head was supported by a chin rest. Participants used their right hand to manipulate a finger movement device placed on a table. For this purpose they placed their index finger and the thumb on two U-shaped metal plates which were mirror-symmetrically interlocked (see the left part of Fig. 1). In order to make the finger movements more comfortable to the participants and to prevent possible exploratory finger movements during the judgments the index and the middle fingers of the right hand were bound together. Auditory stimuli were presented through headphones.

### Stimuli and procedure

All stimuli were presented on a gray background filled with 3,913 black dots of 1.2 mm in size. The dots were randomly distributed across the entire screen before each block of trials. There were three main types of trials – bimodal, visual and haptic. In the bimodal and visual trials the main visual stimulus was composed of a number of black unfilled circles (1.2 mm in diameter) randomly distributed along the defined width and height of a virtual rectangle (cf. the right part of Fig. 1). We refer to this stimulus to as target object hereafter. The width of the target object was always 3.1 cm. Its height, the number of circles, as well as the duration of presentation varied according to the experimental conditions (see Design).

#### Bimodal trials

At the beginning of each trial a fixation cross appeared in the middle of the screen (4 × 4 mm; dark gray). In response to this stimulus participants had to adopt a starting finger posture by moving their fingers apart from each other. A short beep tone and a pair of green circles (3 mm in diameter) were presented when the distance between the fingers exceeded 7.5 cm. This was an obligatory part of the trial. The circles served as movement cursors which had to be placed at the opposed edges of a rectangular target object by moving the fingers to each other. When the distance of the circles to the edges of the target was less than 5 mm the circles disappeared. This was done in order to equalize the visual input between the bimodal and visual trials during the critical period of target appearance. Note that the target object appeared only when the (invisible) cursors approached the edges of the target (i.e. when the center of the circle was less than 2 mm apart from the edge). The purpose of not presenting the target before was to reduce the strong impact of visual information as suggested in^19^ and as was also done in^20^. The correct “grabbing” was also signaled by clicking noise that was included to make the bimodal and haptic trials as comparable as possible. Participants were instructed to maintain this finger position for 1 sec (duration of the clicking noise) and to perform corrective movements when the noise disappeared (i.e., when the circles left the edges of the rectangle). Then the fixation cross reappeared and after adopting the starting finger posture (see above) a second grabbing movement had to be performed. Finally, in response to a blue question mark (5 mm) the participants should indicate which of the target objects was larger by pressing the left (for “first”) or the right (for “second”) key of a computer mouse using their left hand. This judgment was performed while the final finger posture of the second grabbing movement was maintained.

#### Haptic trials

The general procedure for the haptic trials was the same as for the bimodal trials. That is, the participants spread the fingers to adopt a starting position, moved them then together until a certain posture was achieved, spread the fingers again and moved them again together until another posture was achieved, finally a question mark appeared in response to that a judgment was made. The following changes were implemented in the haptic trials. Neither visual target objects nor movement cursors were presented. After the starting finger posture was adopted a German word for “search” appeared at the middle of the screen. The task here was to move the fingers until a certain finger distance was reached at which clicking noise was presented. After the starting finger posture was readopted and the second movement was performed, participants had to indicate which finger distance was larger.

#### Visual trials

No finger movements were executed in the visual trials. The fixation cross was shown for 1100 msec. Following 500 ms after its disappearance the first visual target object was presented together with clicking noise as in the bimodal trials. The second target object was presented in the same way. After the second target object disappeared the size judgment was performed in the same way as in the bimodal trials.

When the participants changed the fingers’ posture of their right hand during the judgments or when the left or the right mouse buttons were pressed during the grabbing phases an error feedback was presented and the trial was repeated.

### Design

In the visual trials, one of the two presented target objects was always the standard stimulus with a constant height of 4.3 cm. The height of the other object (test-stimulus) varied between 2.5 and 6.1 cm in ten steps. In the haptic trials, these values corresponded to the finger distances. The test stimuli in the bimodal trials were the same as in the visual trials. For the standard stimulus, in contrast, a crossmodal conflict was introduced between the visual and the haptic signals. This was accomplished by differently transforming the finger movements into the movements of visual cursors. In one half of trials, a finger distance of 4.9 cm had to be adopted to “grab” the visual target stimulus of 3.7 cm in height (small gain condition hereafter). In the other half of trials, this relation was reversed. That is, the visual size of 4.9 cm corresponded to the haptic distance of 3.7 cm (large gain hereafter). Implementing the conflict this way and presenting both trial types in randomized order should prevent crossmodal recalibration^19^. The standard stimulus was in 50% of the trials the first presented object, in the remaining trials the second (random assignment).

Additionally, there were two reliability level conditions in the bimodal and visual trials. In the high reliability condition the target object consisted of many circles (between 96 and 233 depending on the current rectangle’s height). In the low reliability condition, in contrast, there were only few circles (between 10 and 23). Additionally, the duration of target presentation was 1000 ms in the high reliability and 100 ms in the low reliability condition. Note that this did not change the whole timing of the trial because the clicking noise was always presented for 1 sec. Both manipulations served the same purpose, namely to induce a substantial difference in the reliability of visual target information between both reliability level conditions. We reasoned that decreasing the number of dots alone will not be sufficient to substantially reduce the visual reliability in the low reliability condition.

There were thus seventy different conditions (bimodal: 10 test sizes × 2 gain conditions × 2 reliability conditions + visual: 10 test sizes × 2 reliability conditions + haptic: 10 test sizes). The main experiment included 1,120 trials (sixteen repetitions of each condition) which were distributed over two separate experimental sessions taking place on two different days (In two participants, the repetition factor was not 16 in each bimodal condition but ranged between 14 and 18 due to technical reasons, i.e. due to computer crash during the rest period in one of two sessions). Each session lasted about two hours and included fourteen blocks of forty trials each. The factor modality was held constant within each block of trials, whereas all other conditions were randomly presented in each block. A random presentation of bimodal and unimodal trials would require three different cues signaling each trial type. We assumed that this could confuse the participants and lead to avoidable mistakes and thus decided in favor of the blocked presentation as was also done in a previous related study^20^. In each session, eight blocks including bimodal trials, two blocks including haptic trials and four blocks including visual trials were presented in a random order. Following each block participants could make short breaks. After the seventh block an obligatory rest period of at least 10 minutes was implemented in each session.

At the beginning of each session eighteen practice trials were performed which were not included in the analysis (six for each modality type).

### Main analyses

For each participant and each modality, reliability and gain condition we computed the proportion of trials in which the test stimulus was judged as larger as a function of the test size. These values were then fitted with a psychometric function by using a local model-free fitting procedure^22^. The mean *r*^2^ amounted 0.95 (*SD* = 0.08).

The point of subjective equality (PSE) was determined by identifying the test size at which the psychometric function crosses the 50% point. The just noticeable difference (JND) was calculated as a difference between 50% and the 84% points^20^. To understand how these measures relate to the MLE model consider that sensory information is noisy. If an object is, e.g., 10 cm in size its perceived size usually varies around this value. The probability of these different estimates can be modelled by a bell-shaped Gaussian function (called probability density function) with a mean corresponding to the most likely size (μ) and its standard deviation (σ) relating to signal reliability. The discrimination performance in a 2-AFC task is usually “S”-shaped when the proportion of “test is larger” judgments is plotted against the difference between the test and standard stimuli. This psychometric function is assumed to result from integrating (i.e. summing up) the values of a bell-shaped Gaussian function. Accordingly, the σ parameter can be derived from a difference between the 50% and 84% points of the psychometric function (i.e. JND). The JND corresponds to √2 * σ for a 2-AFC task. The mean of the underlying function (μ) corresponds to the PSE that describes the size of the test stimulus at which the test and the standard stimuli are judged equal (i.e. at which the psychometric function crosses the 50% point). The PSE values are thus indicative of perceptual biases towards one modality in the bimodal conflict trials predicted by the model (see below). For further details see^1^.

Accordingly, the observed variance of the sensory signals was computed using the following formula^19^:

$${{\rm{\sigma }}}_{{\rm{obs}}}^{2}={{\rm{JND}}}^{2}/2.$$

This index of cue uncertainty increases with a decrease in discrimination performance and is crucial for the testing of MLE predictions. In particular, it is used to predict the psychometric function for the bimodal conditions (i.e. the weights and variances) based on the data of the unimodal conditions (see below).

The observed visual weights captured perceptual biases in bimodal conflict trials and were computed as

$${{\rm{w}}}_{{\rm{v}}\_{\rm{obs}}}=({\rm{PSE}}-{{\rm{S}}}_{{\rm{h}}})/({{\rm{S}}}_{{\rm{v}}}-{{\rm{S}}}_{{\rm{h}}})$$

where S_h_ and S_v_ are the finger distance adopted during grabbing and the height of the visual stimulus respectively^20^. This measure basically reflects the deviation of the perceived size of the target stimulus from its visual size and can adopt values between “0” (perceived size = haptic size) and “1” (perceived size = visual size). Thus, the larger the value is the less haptic impact can be inferred. Note that the visual and haptic weights add up to 1. That is, the haptic weights are given by 1 − w_v_obs_. The weights and variances observed in two gain conditions were averaged prior to statistical analyses.

The predicted visual weights for optimal integration were computed according to

$${{\rm{w}}}_{{\rm{v}}\_{\rm{pred}}}=(1/{{{\rm{\sigma }}}^{2}}_{{\rm{v}}\_{\rm{obs}})}/(1/{{{\rm{\sigma }}}^{2}}_{{\rm{v}}\_{\rm{obs}}}+1/{{{\rm{\sigma }}}^{2}}_{{\rm{h}}\_{\rm{obs}}})$$

where σ^2^_v_obs_ and σ^2^_h_obs_ are observed variances in the unimodal visual and haptic conditions respectively^19^. The MLE model thus predicts the relative impact of the visual and haptic signals on the perception of target object in the bimodal trials based on the relative precision (or reliability) of these signals measured in the unimodal trials. The less reliable signal (with larger variance/JND) receives less weight.

The predicted optimal bimodal variances are given by

$${{{\rm{\sigma }}}^{2}}_{{\rm{vh}}\_{\rm{pred}}}={{{\rm{\sigma }}}^{2}}_{{\rm{v}}\_{\rm{obs}}}{{{\rm{\sigma }}}^{2}}_{{\rm{h}}\_{\rm{obs}}}/({{{\rm{\sigma }}}^{2}}_{{\rm{v}}\_{\rm{obs}}}+{{{\rm{\sigma }}}^{2}}_{{\rm{h}}\_{\rm{obs}}}).$$

The MLE model thus states that the variance in the bimodal trials should be lower than the variances measured in each of the unimodal conditions^19,20^. As an index of cue uncertainty we used the standard deviation (σ = √σ^2^) rather than the variance for further analyses because the units of σ are more easily interpretable as the units of σ^2^ (cf^19^).

Statistically optimal integration is given if the observed bimodal uncertainties are not significantly different from the predicted ones and are lower than each of the unimodal uncertainties. Also, the observed weights should not be different from the predicted weights.

### Additional analyses

A JND measured as a difference between 50% and the 84% points of the psychometric function corresponds to one standard deviation of the underling probability density distribution which is assumed to have a Gaussian shape as mentioned earlier^1^. Accordingly, the JND computed this way is important for the derivation of optimal weights and uncertainties. It is thus critical that this type of JNDs adequately captures participants’ judgment behavior to reliably test for optimal integration.

As indicated by Fig. S1, the discrimination performance of several participants in at least one of the critical conditions was rather low so that the 84% point of the psychometric functions was not reached. To be on the safe side we pursued a very conservative approach and excluded the data of these participants from the main analyses.

To approve that the results hold for the whole sample, we also run additional analyses including all participants. In particular, we calculated another type of JND (JND’) identifying the test heights corresponding to the 40% and 60% points of the psychometric function and then halving the difference between these values. These JNDs as well as PSEs were statistically compared across the critical conditions. We also rerun the main analyses including the originally computed JNDs of all participants. The results of these analyzes can be found in the section “Supplementary materials”. They justified the results of the main analyzes and suggested that these results are representative for the whole sample of the recruited participants.

The data have been made publicly available (https://osf.io/q7bth/).

## Results

**Figure 2:**
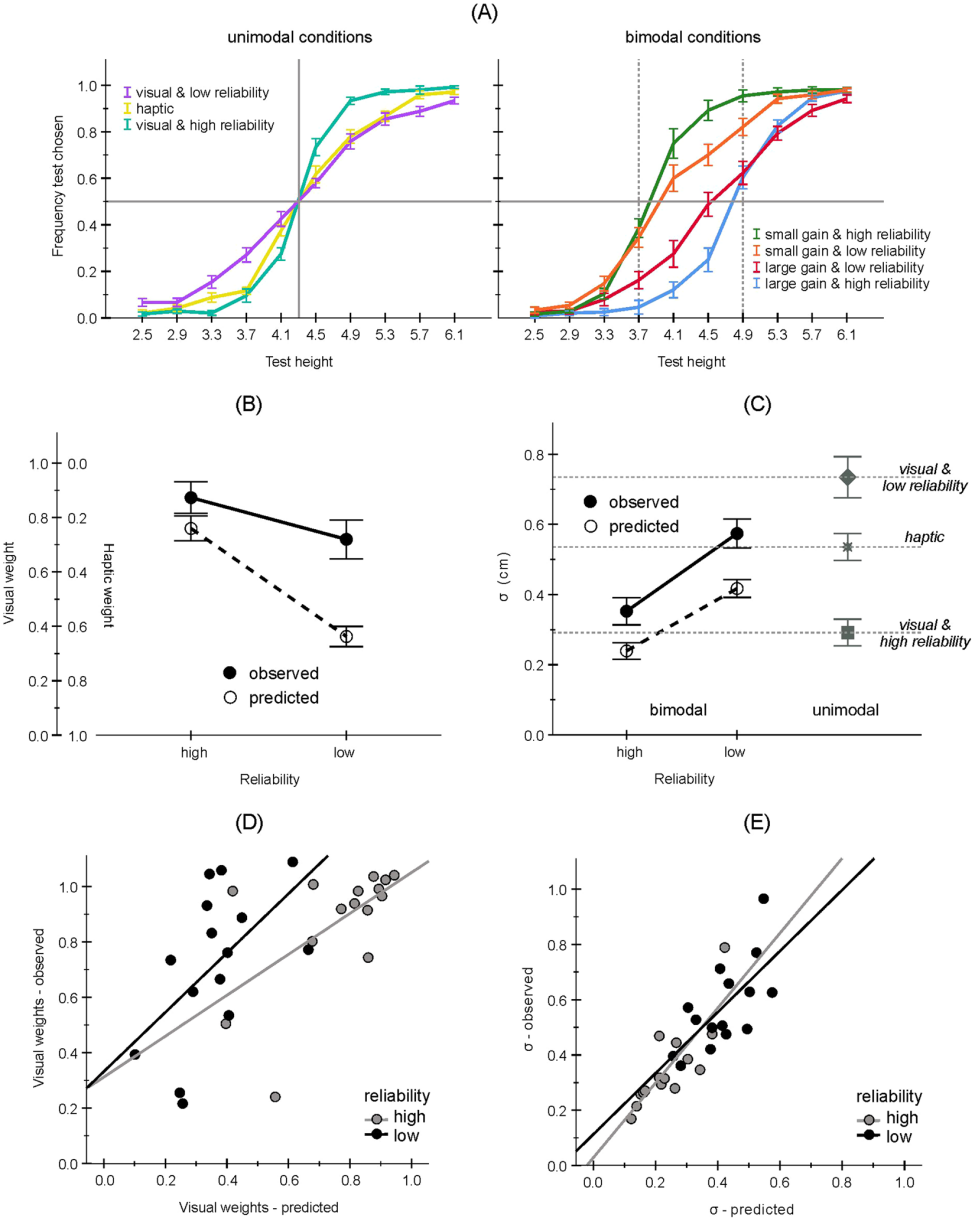
(**A**) Proportion of trials in which the test stimulus was judged as larger against the size of the test stimulus. In the unimodal conditions, the size of the standard stimulus was 4.3 cm. In the bimodal conflict trials, the visual/haptic standard stimulus was either 3.7/4.9 cm (small gain) or 4.9/3.7 cm (large gain). (**B**) Predicted and observed weights. (**C**) Predicted and observed variabilities (i.e. standard deviations). (**D**) Relationship between individual observed and predicted visual weights. (**E)** Relationship between individual observed and predicted variabilities. Error bars indicate standard errors. The data of 15 participants included in the main analyzes are shown (see Methods).

The upper part of Fig. 2(A) shows the mean proportions of trials in which the test stimulus was judged as larger as a function of its size in the unimodal and bimodal conditions. The data of a subsample of participants which were included in the main analyses is illustrated (N = 15, see also Figs. S1 and S2 for the data of all participants). As expected, the slope of the indicated psychometric functions is smaller when the reliability of the visual stimulus decreases. Moreover, in the bimodal conditions the functions are shifted from the visual standard stimulus towards the haptic stimulus and this bias is larger when the visual stimulus is less reliable.

### Weights

The perceptual biases were captured by the visual weights shown in Fig. 2(B). An analysis of variance (ANOVA) including prediction and reliability level as within-participants factors revealed significant main effects for both factors, *F*(1, 14) = 23.46, *p* < 0.001, *η*_*p*_^2^ = 0.626, and *F*(1, 14) = 112.13, *p* < 0.001, *η*_*p*_^2^ = 0.889, as well as a significant interaction, *F*(1, 14) = 24.79, *p* < 0.001, *η*_*p*_^2^ = 0.639. Both the observed and the predicted visual weights were smaller in the low reliability condition than in the high reliability condition, *t*(14) = 4.10, *p* = 0.001, and *t*(14) = 11.70, *p* < 0.001. The predicted weights were generally smaller than the observed weights, *t*(14) = 2.32, *p* = 0.036 (low noise) and *t*(14) = 6.00, *p* < 0.001 (high noise). This difference was, however, more pronounced for the lower level of reliability (see significant interaction above). All weights were also significantly different from one, all *p*s < 0.046.

Moreover, the individual predicted weights correlated significantly with the observed weights in both reliability level conditions, *r* = 0.582, *p* = 0.023 (high reliability) and *r* = 0.554, *p* = 0.032 (low reliability, see Fig. 2D).

### Variability

Figure 2(C) illustrates the mean observed and predicted standard deviations (*σ***)**. An ANOVA including bimodal conditions and prediction and reliability level as factors revealed significant main effects for both factors, *F*(1, 14) = 32.00, *p* < 0.001, *η*_*p*_^2^ = 0.696, and *F*(1, 14) = 97.53, *p* < 0.001, *η*_*p*_^2^ = 0.874. The interaction was not significant, *F*(1, 14) = 2.84, *p* = 0.114, *η*_*p*_^2^ = 0.168. The observed variability increased with a decrease in visual reliability as predicted. However, it was generally larger than predicted.

The observed bimodal variability in the high reliability condition was also larger than in the corresponding visual unimodal condition, *t*(14) = 2.19, *p* = 0.046, but lower than in the haptic unimodal condition, *t*(14) = 4.57, *p* < 0.001. The observed bimodal variability in the low reliability condition was not significantly different from the haptic unimodal condition, *t*(14) = 1.26, *p* = 0.230, but was lower than in the corresponding visual unimodal condition, *t*(14) = 2.78, *p* = 0.015.

Moreover, the predicted standard deviations correlated significantly with the observed standard deviations in both reliability level conditions, *r* = 0.817, *p* < 0.001 (high reliability) and *r* = 0.683, *p* = 0.005 (low reliability; see Fig. 2E).

## Discussion

The goal of the present study was to examine whether perceptual biases observed in the context of a task including virtual interactions with a distant object are due to statistically optimal integration of body-related and visual signals relating to a common external object. The results were in line with qualitative predictions of the MLE rule. The perceived size of the target object was closer to its more reliable, i.e. visual, size and this bias decreased when the visual stimulus became less reliable. Moreover, the predictive value of the model was also suggested by the systematic relation between observed and predicted measures of bias and variability on the individual level.

However, the model predicted a lower impact of vision on average especially for the less reliable stimulus. Moreover, the observed precision of discrimination performance was systematically lower than predicted by the model. Also, the bimodal precision was not higher than in each unimodal condition. These results entail two basic interpretations. First, the visual and haptic signals were not integrated. Second, the signals were integrated according to the reliability-based weighting, but there were some additional factors that the model did not capture.

Consider that the variability in the bimodal trials of the high reliability condition was close to the variability of the visual unimodal trials of the same reliability condition but substantially lower than in the haptic trials (see Fig. 2C). In the low reliability condition, in contrast, the bimodal variability was comparable to the haptic variability, but was lower than the unimodal visual variability. This could indicate that the participants applied a cue switching strategy and used the visual/haptic cues more when the visual reliability was high/low. In other words, the more reliable unimodal signal could have been used for judgment rather than a multimodal percept. The observed biases, however, speak against this possibility. A switch to the haptic signal in the bimodal low reliability condition should result in a size perception which is close to the haptic size. This was definitely not the case (cf. Fig. 2A,B).

A similar variability pattern was previously repeatedly observed by Debats and colleagues in a cursor-control task, where hand movements and their visual effects occur in separate spatial planes^6,7,9^. The authors nevertheless argued in favor of sensory integration. The deviations from model predictions were discussed in the context of variance not included by the model and possible violations of model assumptions. In a similar vein, model deviations relating to the variability we observed could arise from additional sources of noise inherently present in the current experimental paradigm. This additional variability can be present on several levels, including stimuli, decisions, motor behavior or motivation. Consider, e.g., that there were many blocks including bimodal trials as compared with blocks including unimodal trials (16 × bimodal, 8 × visual, 4 × haptic). This could have increased/decreased the motivation of the participants when the rare/frequent blocks were announced which might explain an increase of variability in bimodal compared to unimodal conditions on top of reliability-based weighting. In other words, the reliabilities could have been overestimated in the unimodal trials as compared to the bimodal ones.

Debats and colleagues also reported that under certain conditions the weights observed in the cursor-control task did not match the weights predicted by statistically optimal integration (see Exp.1 in^6^). This was attributed to specific contextual factors which can reduce or promote trust in a certain cue and thus affect the bias in addition to purely reliability-based weighting. This argument is also applicable to the present paradigm of virtual object-oriented actions. For example, the general overweighting of visual information could originate from visual cursors, or more generally from visual movement effects, which are present in the bimodal but absent in the unimodal trials. The impact of these stimuli could have been twofold. First, they could generally bias the participant to strongly weight the visual target information and to ignore the fingers. Second, these stimuli also provided some visual clues about the object size (though not as exact as the target and fingers) which could have been directly used for the judgments in the bimodal trials. These factors could have contributed to the biases measured in the bimodal trials and caused systematic deviations from the MLE predictions. Alternatively, visual information could a-priori receive more weight when body-related and environmental effects are spatially separated^23^. Despite these possible influences unrelated to the reliability-based weighting the observed impact of the reliability manipulation on the weights can still be considered as indication of optimal integration.

The task we used shares several crucial features with tasks used previously, e.g., in tool-use studies or in the research on actions’ effects in visual perception^15^. Moreover, the MLE rule has been approved in several multimodal setups. Accordingly, we believe the present results are potentially generalizable to a wide range of experimental situations. However, each paradigm has its own specifics that affect the results and that should be taken into account. In addition to the factors we already mentioned, one might object that the present task is rather unnatural and unfamiliar to the participants and that this caused deviations from the model predictions. It is, thus important to compare the current results to the results from experiments with more natural object interactions in future studies. This can be achieved, e.g., when virtual reality techniques are combined with force-feedback devices. Moreover, it could be fruitful to extend the MLE model to a measure of sensory coupling (so called coupling prior) as was done in the previous related research^6–9^. This will enlarge the scope of the model to the impact of vision on body perception.

To sum up, the present study indicates a compensatory mechanism in virtual interactions with distant objects which tries to reduce sensory noise similar to reliability-based integration of multimodal signals. However, whether this mechanism in fact reflects statistically optimal integration or rather selective using of different sensory cues remains to be determined. In spite of this, this mechanism might be responsible for manifold effects of action on visual perception observed in diverse interactions with external objects. We thus suggest that the impact of an action on perception is mediated by body-related consequences of that action which inform, alike other senses, about environmental characteristics.

## Supplementary information

Supplementary Information

## Data Availability

The data have been made publicly available via the Open Science Framework and can be accessed at https://osf.io/q7bth/.
