# Supplementary material for: Multisensory integration in virtual interactions with distant objects
Source: Sci Rep. 2019 Nov 22;9:17362. doi: 10.1038/s41598-019-53921-9 (PMC6874595; doi:10.1038/s41598-019-53921-9)
Supplement: Supplementary file 1 — Supplementary Information [file 41598_2019_53921_MOESM1_ESM.pdf]

Supplementary Information for

Multisensory integration in virtual interactions with distant objects

Wladimir Kirsch* and Wilfried Kunde

Department of Psychology, University of Würzburg, Germany

*Corresponding author: kirsch@psychologie.uni-wuerzburg.de

Content:

Figure S1

Additional analyses

Figure S2

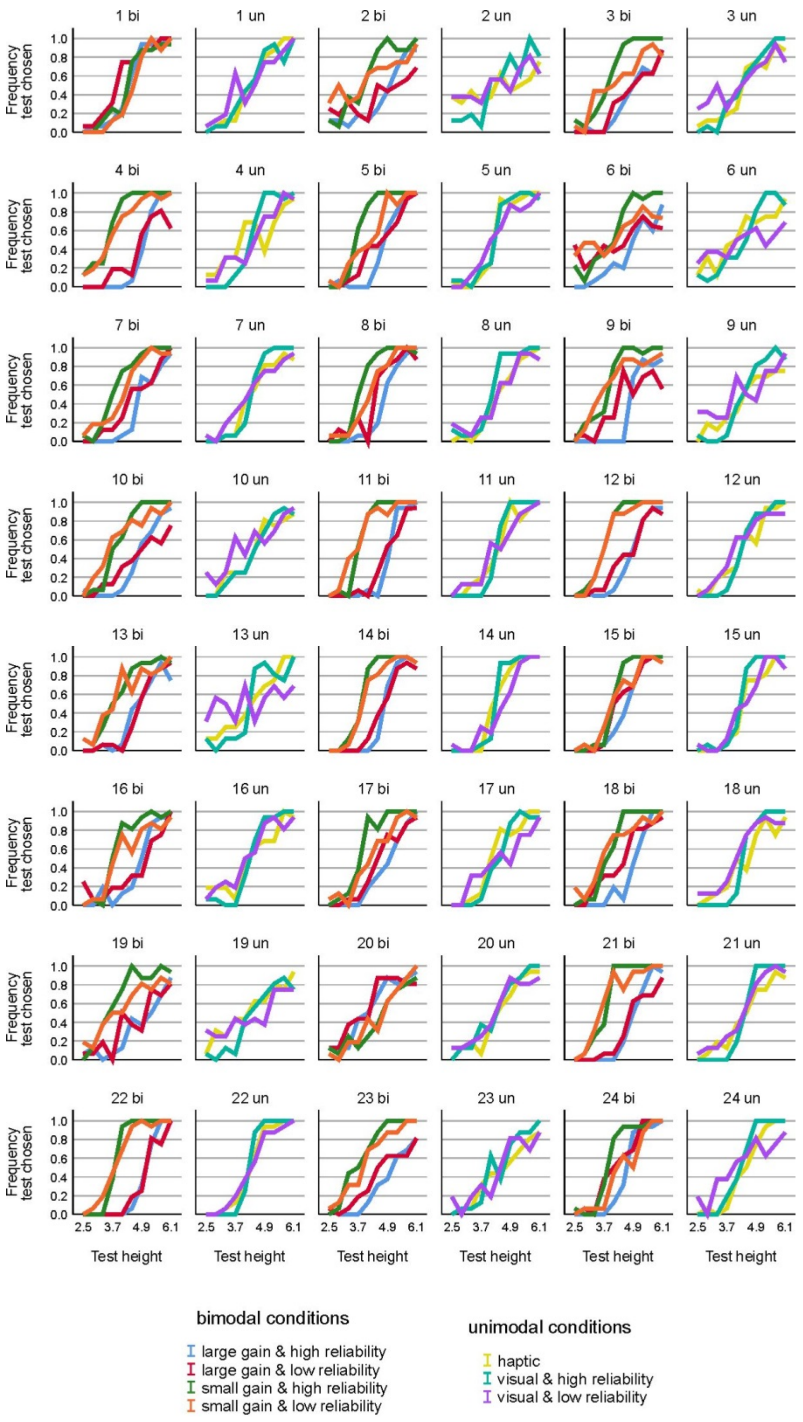

Figure S1. *Proportion of trials in which the test stimulus was judged as larger against the size of the test stimulus. The data of all participants and all conditions are shown. Digits indicate participants' number, abbreviations "bi" and "un" stand for bimodal and unimodal conditions respectively.*

Additional analyses

To ensure that the results of main analyses hold for the entire sample of participants we have recruited some additional analyses were run (see also Methods).

(1) PSEs. The bimodal PSE of the high reliability condition was significantly larger than the PSE of the low reliability condition when the gain was large, $t(23) = 3.76$, $p = .001$, and it was significantly smaller when the gain was small, $t(23) = 2.90$, $p = .008$ (see Fig. S2, B for means). This result confirms the modulation of the empirical bimodal visual weights with varying reliability observed in the main analyses.

(2) JND'. The JNDs of all high reliability conditions were smaller than the JNDs of the low reliability conditions, all ps < .001 (see Fig.S2, C for means). Moreover, the bimodal JNDs in the high reliability conditions were not significantly different from the unimodal high reliability condition, t(23) = 1.16, p = .259 (large gain), and t(23) = .77, p = .450 (small gain), but were lower than in the haptic unimodal condition, t(23) = 3.01, p = .006 (large gain), and t(23) = 3.87, p = .001 (small gain). The bimodal JNDs in the low reliability conditions were not significantly different from the haptic unimodal condition, t(23) = .32, p = .752 (large gain), and t(23) = .88, p = .389 (small gain), but were lower than in the visual unimodal condition with the lower reliability, t(23) = 2.24, p = .035 (large gain) and t(23) = 3.25, p = .004 (small gain). These results confirm the results of the main analyses. In particular, there were no indications that the variability of the bimodal conditions was lower than in each unimodal condition. Rather, the bimodal variability approached the variability of the more reliable signal like in the main analyses.

(3) Main analyses. Including all participants in the main analyses did not change the results substantially. Four marginal deviations were observed. First, the difference between predicted and observed standard deviations was now significantly larger for the low reliability condition than for the high reliability condition, $F(1, 23) = 8.24$, $p = .009$, $\eta_p^2 = .264$. Second, the bimodal variability in the high reliability condition was not significantly different from the variability in the unimodal visual condition, $t(23) = .02$, $p = .984$. Third, the observed bimodal visual weight in the high reliability condition was now not significantly different from one, $t(23) = 1.67$, $p = .108$. Fourth, the individual correlation between the predicted and observed weights was only marginally significant in the high reliability condition, $r = .391$, $p = .059$.

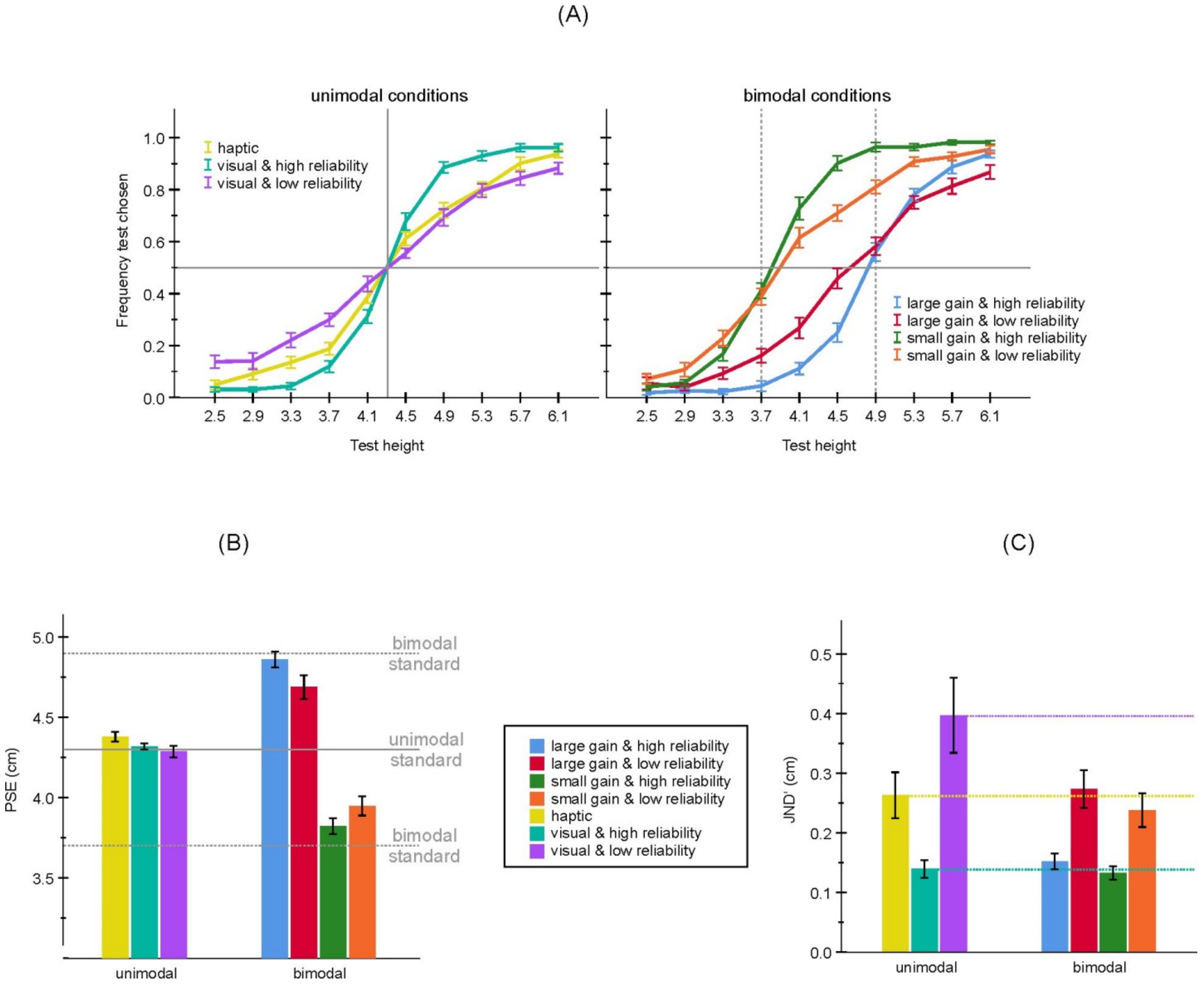

Figure S2. *(A) Mean proportion of trials in which the test stimulus was judged as larger against the size of the test stimulus. (B) Mean PSEs of all conditions. (C) Mean JND' for each condition. Error bars are standard errors. The data of all participants are shown (N=24).*
